# The Association of Cognitive Status and Post-Operative Opioid Prescribing in Older Adults

**DOI:** 10.1097/AS9.0000000000000320

**Published:** 2023-08-21

**Authors:** Christina L. Shabet, Mark C. Bicket, Emilie Blair, Hsou Mei Hu, Kenneth M. Langa, Mohammed U. Kabeto, Deborah A. Levine, Jennifer Waljee

**Affiliations:** From the * Department of Surgery, University of Michigan Medical School, University of Michigan, Ann Arbor; † Department of Anesthesiology, University of Michigan, Ann Arbor; ‡ Michigan Opioid Prescribing Engagement Network (Michigan OPEN), Institute for Healthcare Policy and Innovation, Ann Arbor; § Department of Internal Medicine and Cognitive Health Services Research Program, University of Michigan, Ann Arbor; ∥ Department of Surgery, Section of Plastic Surgery, University of Michigan, Ann Arbor; ¶ Department of Internal Medicine, University of Michigan, Ann Arbor; # Institute for Social Research, University of Michigan, Ann Arbor; ** VA Center for Clinical Management Research, Ann Arbor; †† Center for Health Outcomes and Policy, University of Michigan, Ann Arbor, MI.

**Keywords:** cognitive impairment, cognitive status, dementia, opioid use, postoperative, surgical outcomes

## Abstract

**Objective:**

To examine the differences in opioid prescribing by cognitive status following common elective surgical procedures among Medicare beneficiaries.

**Background:**

Older individuals commonly experience changes in cognition with age. Although opioid prescribing is common after surgery, differences in opioid prescribing after surgery by cognitive status are poorly understood.

**Methods:**

We conducted a retrospective analysis of patients ≥65 years participating in the Health and Retirement Study (HRS) linked with Medicare claims data who underwent surgeries between January 2007 and November 2016 and had cognitive assessments before the index operation. Cognitive status was defined as normal cognition, mild cognitive impairment (MCI), or dementia. Outcomes assessed were initial perioperative opioid fill rates, refill rates, and high-risk prescriptions fill rates. The total amount of opioids filled during the 30-day postdischarge period was also assessed. Adjusted rates were estimated for patient factors using the Cochran-Armitage test for trend.

**Results:**

Among the 1874 patients included in the analysis, 68% had normal cognition, 21.3% had MCI, and 10.7% had dementia. Patients with normal cognition (58.1%) and MCI (54.5%) had higher initial preoperative fill rates than patients with dementia (33.5%) (*P <* 0.001). Overall, patients with dementia had similar opioid refill rates (21%) to patients with normal cognition (24.1%) and MCI (26.5%) (*P =* 0.322). Although prior opioid exposure did not differ by cognitive status (*P =* 0.171), among patients with high chronic preoperative use, those with dementia had lower adjusted prescription sizes filled within 30 days following discharge (281 OME) than patients with normal cognition (2147 OME) and MCI (774 OME) (*P <* 0.001; *P =* 0.009 respectively). Among opioid-naive patients, patients with dementia also filled smaller prescription sizes (97 OME) compared to patients with normal cognition (205 OME) and patients with MCI (173 OME) (*P <* 0.001 and *P =* 0.019, respectively).

**Conclusions:**

Patients with dementia are less likely to receive postoperative prescriptions, less likely to refill prescriptions, and receive prescriptions of smaller sizes compared to patients with normal cognition or MCI. A cognitive assessment is an additional tool surgeons can use to determine a patient’s individualized postoperative pain control plan.

## Abstract 2

Mini Abstract

To examine perioperative opioid fill rates, refill rates, and high-risk prescription fill rates according to cognitive status, we conducted a retrospective analysis of older patients (65+ years) who underwent surgery and previous cognitive assessments. Patients with dementia were less likely to receive initial opioid prescriptions, refills, and high-risk prescription fill rates than those with normal cognition.

## INTRODUCTION

Up to 30% of older adults in the National Poll on Healthy Aging report receiving a prescription for opioid pain medication.^1^ Older adults (65+) are more likely to develop new persistent use of opioids than younger adults, and high-risk opioid prescribing is common in older adults with more than 500,000 Medicare Part D beneficiaries receiving high amounts of opioids in 2016.^2^ Opioid prescribing after surgery is common and an independent risk factor for prolonged opioid use among both opioid-naive and opioid-exposed individuals.

Despite this, much less is known regarding the patterns of opioid prescribing among older individuals by cognitive status. Millions of older Americans have mild cognitive impairment (MCI), defined as an early-stage memory loss or other cognitive ability loss in patients who maintain the ability to independently perform most activities of daily living (ADLs), or dementia, defined as a decline in memory and other cognitive abilities severe enough to interfere with daily life.^3^ Alzheimer’s is the most common form of dementia; it is a progressive neurodegenerative disease beginning with mild memory loss and progressing to loss of ability to converse and/or respond to one’s environment.^4^ The key difference between MCI and dementia is the preservation of independence in ADLs and instrumental activities of daily living and the lack of significant impairment in both social and occupational functioning. It is important to identify initial symptoms of MCI in older adults to implement a care plan including aerobic exercise and daily mental activity, to control for other risk factors, and to assess for the risk of progressing to dementia.^5^ Prior studies have shown that patients with MCI and dementia may have a higher risk of inappropriate drug use than patients with normal cognition due to difficulty with medication self-management.^6^ A 2018 systematic review on medication management issues in dementia attributed this inappropriate drug use to the decline in cognitive ability affecting patients with dementia’s ability to plan, execute, manage, and organize medicine-related tasks.^7^ However, little is known about the prevalence and risks of opioid prescribing to older adults with cognitive impairment after surgery, who may be particularly vulnerable to the risks of opioid analgesics during the postoperative period.^6^ This topic is particularly relevant given the rise if the number of older adults in the US population, which is expected to reach 80.8 million in 2040 and 94.7 million by 2060, making up 25% of the total US population.^8^

In this retrospective cohort study, we examined the relationship between cognitive status (normal cognition, MCI, and dementia) and opioid prescribing following common elective surgical procedures. We specifically examined the rate of initial postoperative fill, the rate of postoperative refill, the total amount of opioid prescribed in the 30 days following surgery, and the rate of high-risk prescribing among adults 65 years and older participating in the nationally representative Health and Retirement Study (HRS) with linked Medicare data and repeated cognitive assessments. This dataset presents the unique opportunity to examine cognitive impairment levels paired with claims data, permitting a higher level of granular assessment. We hypothesized that patients with dementia are less likely and patients with MCI are as likely to receive opioid prescriptions following surgery compared to cognitively normal patients.

## DATA AND METHODS

### Study Population and Data Source

We analyzed data from participants in the nationally representative HRS who have linked Medicare claims data, including Medicare Parts A, B, and D from January 2006 to November 2016 based on Current Procedural Terminology Fourth Edition (CPT-4) codes (Supplemental Table S1, http://links.lww.com/AOSO/A233, which demonstrates CPT-4 codes included in the analysis). The patient population analyzed within the combined data set were patients who had undergone surgeries between January 2007 and November 2016, were 65 years or older, and had cognitive assessments before the index operation. We examined the following surgery types: abdominal, breast, cardiac, extremity, gynecologic, head/neck/face, hip replacement (total or partial), knee replacement, neurosurgery, shoulder surgery, soft tissue, spine, thoracic, urologic, and vascular. For patients who had multiple surgeries during the study period (N = 966; 51.5%), the first surgery was used for analysis. Elective and nonelective surgical status as well as inpatient and outpatient procedures were distinguished using claims data. The research protocol was approved by the University of Michigan Institutional Review Board.

### Exclusion Criteria

Patients were excluded from the dataset if they were without continuous Medicare parts A, B, or D 1 year before admission for surgery and 30 days after discharge. Additionally, patients who had longer than 30 days of stay during the index hospital stay, as well as those who were not discharged home or died within 30 days of discharge, were excluded from our dataset. Patients who had another surgery within 30 days after discharge were excluded; these patients were excluded to avoid counting opioid prescriptions filled for the subsequent surgery within 30 days following the index surgery.

### Outcomes Assessed

Our outcomes included the percentage of patients who filled initial perioperative opioid prescriptions 30 days before admission for surgery up to 3 days after discharge. Opioid prescription data were obtained from Medicare Part D claims based on national drug codes. We examined the refill rate and proportion of patients with high-risk prescriptions. High-risk prescribing was defined as patients who had overlapping opioid prescriptions, overlapping opioid and benzodiazepine prescriptions, daily Oral Morphine Equivalent (OME) ≥100, a long-acting opioid prescription, and/or if the patient filled opioid prescriptions from more than one prescriber.^9^ The total number of opioid prescriptions was the sum of all prescriptions filled from the discharge date up to the 30-day post-discharge period. The size of a prescription was measured as OMEs, the product of strength, quantity, and a factor that converted different types of opioids into the equivalent OME amount.^10^

### Exposures Assessed

Cognitive status was categorized as normal cognition, MCI, and dementia. Trained HRS interviewers administered cognitive function tests biennially in-person or by telephone using the Modified Telephone Interview for Cognitive Status (TICS-m), a global cognition test patterned after the Mini-Mental State Examination.^11^ The TICS-m assesses global cognition, learning, and memory (scores range, 0–27). Higher scores indicate better performance. Among older adults, telephone measurement of global cognition, learning, and memory, compared to in-person measurement, is comparable, reliable, and precise.^11,12^ The TICS-m is validated as a cognitive screening instrument and designed for use in population-based studies. Proxy respondents had separate assessments. At each interview, the HRS participant was classified as having normal cognition, MCI, or dementia using methods and validated cut-points based on in-depth, in-home, neuropsychological, and clinical assessments, as well as expert clinician adjudication from the aging, demographics, and memory study, an HRS dementia sub-study.^13,14^ Respondents represented by a proxy were assessed using a separate 11-point scale of the proxy’s assessment of the patient’s memory, the respondent’s limitation in 5 instrumental (ADLs), and the interviewer’s assessment of the patient’s difficulty completing the interview due to cognitive impairment. Proxy respondents scoring 6–11 were designated as having dementia and those scoring 3–5 were designated as MCI.^15^

Patients with preoperative opioid exposure were those who filled one or more prescriptions between 365 days and 31 days before admission for surgery. We used clustering analysis to identify patients who had a similar pattern of opioid use based on 4 attributes: total amount of opioids (in OMEs), duration in months, continuity in months, and recency in months.^16,17^ Using the K-medians clustering algorithm, we categorized opioid-exposed patients into 3 groups (low-remote intermediate, median-recent intermediate, and high-chronic) where the attributes had the closest within-group medians and largest between-group medians. Patients who had no opioid prescriptions filled during the same period were classified as opioid naive.

### Patient Characteristics

Preoperative opioid exposure was assessed based on all opioid prescriptions filled between 365 days and 31 days before admission for surgery. Patients were grouped into 4 clusters based on 4 attributes: Total OME, duration in month, continuity in month, and recency in month. A machine learning algorithm, K-median, was used to identify the 3 clusters (low, remote intermittent; medium, recent intermittent; high, chronic), and a fourth cluster, naïve, who were patients who had no opioid prescription filled during the same period (Supplemental Table S2, http://links.lww.com/AOSO/A234, which demonstrates the attributes of patient groups with regards to their preoperative opioid exposure within 1 year before surgery). High-risk prescribing and readmission were assessed based on cognitive status as well.

Patient demographics assessed in this study included age, gender, race, and ethnicity. Self-reported social support factors included marital status and proximity to and having an adult daughter. The socioeconomic factors assessed were education, wealth, and income. Clinical factors included from the HRS survey were tobacco use, depressive symptoms using Center for Epidemiologic Studies Depression Scale 8, and functional limitations before operation.

Charlson Comorbidity Index, history of mental health disorders, and pain disorders were defined using diagnosis codes in claims within 1 year before the surgical admission. Based on the Clinical Classifications Software (CCS) for International Classification of Diseases (ICD)-9-CM and ICD-10-CM diagnostic codes, the presence of anxiety (CCS 651), depression (CCS 657), and psychosis (CCS 659) were identified.^18^ Pain disorders (arthritis, back, neck, and other) were assessed (Supplemental Table S3, http://links.lww.com/AOSO/A235, which demonstrates specific ICD-9 and ICD-10 codes for pain diagnoses). Surgery types, including abdominal, breast, cardiac, extremity, gynecologic, head/neck/face, hip replacement, knee replacement, neurosurgery, shoulder surgery, soft tissue, spine, thoracic, urologic, and vascular surgery, were included in the analysis.

### Analysis

The comparison of patient characteristics among 3 groups of cognitive status was tested using *χ*^2^ tests for categorical variables and analysis of variance for continuous variables. A logistic regression was used to model the association between binary outcomes (filling initial perioperative opioid prescriptions, refills, and having high-risk prescriptions within 30 days), cognitive status, and preoperative opioid exposure while controlling the differences in patient characteristics. A linear regression with robust standard errors based on the Huber-White sandwich estimators was performed to evaluate the relationship between the total amount filled within the postdischarge 30 days, cognitive status and each of the family support variables (geographic proximity to adult children, having adult daughter, or marital status). The interaction term between cognitive status and preoperative opioid exposure was evaluated. Other patient characteristics included in the models were surgery type, elective surgery, inpatient surgery, amount of perioperative opioid, filled initial prescription, age, sex, race/ethnicity, income, Charlson Comorbidity Index, functional limitations, Center for Epidemiologic Studies Depression Scale depressive symptoms, tobacco use, mental health disorders, pain disorders, and surgery year. A final parsimonious model was selected that included clinically relevant variables and variables with a *P*-value <0.3. Model fit was assessed using C-statistics. All data analyses were performed using SAS version 9.4 (SAS Institute Inc., Cary, NC) and Stata version 14.1 (StataCorp, College Station, TX). A two-sided *P <* 0.05 was considered to be statistically significant.

## RESULTS

**FIGURE 1.**
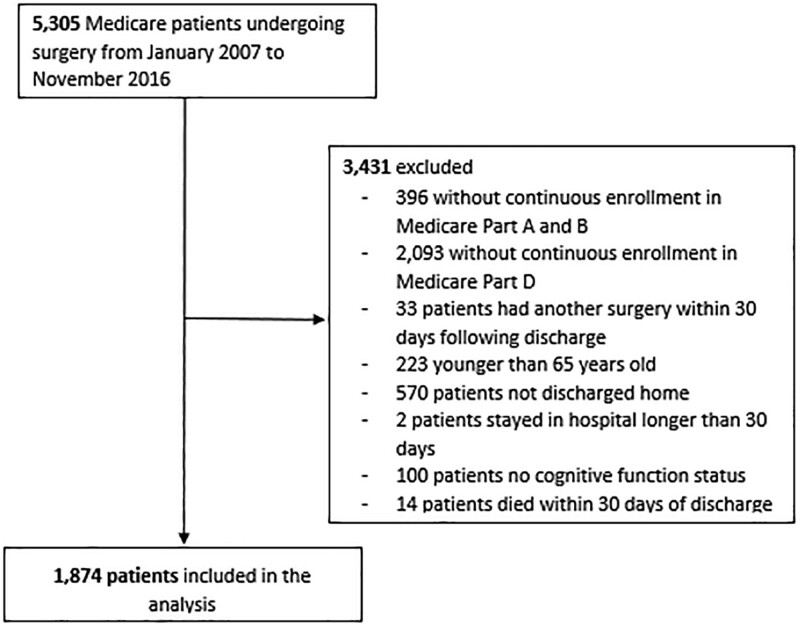
Study population construction.

We identified 5305 Medicare beneficiaries who underwent surgery from January 2007 to November 2016. After applying inclusion and exclusion criteria within this population, 1874 patients were included in the analysis (**Fig. 1**). Of these patients, the mean age was 76.1 and 61.5% of patients were female (**Table 1**). The majority of patients in the cohort analyzed identified their race as non-Hispanic White (77.7%). Opioid-naive patients make up the majority of patients in the analysis at 59.4%, with 11.8% falling into the low/remote intermittent category, 21.0% in the median/recent intermittent, and 7.7% in the high/chronic category of preoperative opioid exposure. The median number of months from cognitive assessment to admission for surgery was 8 months (with an interquartile range (IQR) of 1–5 months).

**TABLE 1.** Patient Characteristics by Cognitive Status (n=1,874)*

	All		Normal Cognitive Function (N = 1274)		Mild Cognitive Impairment (N = 400)		Dementia (N = 200)		
	Number	%	Number	%	Number	%	Number	%	*P* value
Preoperative opioid exposure									0.171
Opioid naïve	1113	59.4%	779	61.1%	220	55.0%	114	57.0%	
Low, remote intermittent	222	11.8%	152	11.9%	51	12.8%	19	9.5%	
Median, recent intermittent	394	21.0%	252	19.8%	96	24.0%	46	23.0%	
High, chronic	145	7.7%	91	7.1%	33	8.3%	21	10.5%	
Elective surgery	1709	91.2%	1166	91.5%	357	89.3%	186	93.0%	0.239
Inpatient	639	34.1%	477	37.4%	128	32.0%	34	17.0%	<0.001
Age: mean (SD)	76.1 (7.3)		74.8 (6.5)		77.9 (7.5)		81.1 (8.5)		<0.001
Sex: female	1152	61.5%	793	62.2%	228	57.0%	131	65.5%	0.079
Race/ethnicity									<0.001
Non-Hispanic White	1457	77.7%	1101	86.4%	254	63.5%	102	51.0%	
Non-Hispanic Non-white	250	13.3%	105	8.2%	79	19.8%	66	33.0%	
Hispanic	167	8.9%	68	5.3%	67	16.8%	32	16.0%	
Education									<0.001
Less than high school	557	29.7%	213	16.7%	211	52.8%	133	66.5%	
High school graduate	626	33.4%	489	38.4%	95	23.8%	42	21.0%	
Some college or more years	691	36.9%	572	44.9%	94	23.5%	25	12.5%	
Income (quartiles)									<0.001
≤14,644	466	24.9%	197	15.5%	158	39.5%	111	55.5%	
14,645–29,504	468	25.0%	307	24.1%	107	26.8%	54	27.0%	
29,505–57,300	471	25.1%	358	28.1%	90	22.5%	23	11.5%	
>57,300	469	25.0%	412	32.3%	45	11.3%	12	6.0%	
Marital status/living arrangement									<0.001
Married/partner	1037	55.3%	765	60.0%	200	50.0%	72	36.0%	
Unmarried/living with other	224	12.0%	113	8.9%	70	17.5%	41	20.5%	
Unmarried/living alone	613	32.7%	396	31.1%	130	32.5%	87	43.5%	
Have an adult daughter	1412	75.3%	955	75.0%	300	75.0%	157	78.5%	0.549
Tobacco use	506	27.0%	339	26.6%	115	28.8%	52	26.0%	0.663
Charlson Comorbidity Index: mean (SD)	3.2 (2.9)		2.8 (2.7)		3.6 (2.9)		4.5 (3.2)		<0.001
Functional limitations, mean (SD), number	1.5 (2.7)		0.7 (1.6)		1.8 (2.7)		5.7 (4.1)		<0.001
CES-D depressive symptoms									<0.001
0	743	39.6%	571	44.8%	120	30.0%	52	26.0%	
1–4	889	47.4%	566	44.4%	214	53.5%	109	54.5%	
5–8	242	12.9%	137	10.8%	66	16.5%	39	19.5%	
Mental health disorders									
Anxiety	225	12.0%	145	11.4%	52	13.0%	28	14.0%	0.450
Depression	296	15.8%	172	13.5%	66	16.5%	58	29.0%	<0.001
Psychosis	57	3.0%	17	1.3%	17	4.3%	22	11.0%	<0.001
Pain disorders									
Arthritis	1505	80.3%	1006	79.0%	323	80.8%	176	88.0%	0.011
Back	738	39.4%	508	39.9%	165	41.3%	65	32.5%	0.096
Neck	308	16.4%	215	16.9%	67	16.8%	26	13.0%	0.382
Other pain disorders	794	42.4%	505	39.6%	192	48.0%	97	48.5%	0.002

*The CMS cell size suppression policy stipulates that no cell containing a value of 1–10 can be reported directly. The following were excluded from the table due to this policy:

Number and percent of patients by surgery type; positive diagnosis of adjustment, suicide, personality, disruptive, other mental health condition or substance and drug use; the highest group of Netwealth and one of the categories of geographic proximity to children among dementia patients had 10 or less cases; and race and education were further collapsed to be 3 categories due to one cell <10 cases.

CES-D indicates Center for Epidemiologic Studies Depression Scale; CMS, Centers for Medicare & Medicaid Services.

**Table 1** presents patient characteristics by preoperative cognitive status: normal cognition (68%, n = 1274), MCI (21.3%, n = 400), and dementia (10.7%, n = 200) categories. Preoperative opioid exposure did not significantly differ between normal cognition, MCI, and dementia (*P =* 0.171). The majority of patients with dementia (52.0%) underwent a minor surgery, soft tissue surgery while relatively fewer dementia patients (14.5%) had a major surgery, such as abdominal surgery. Surgery types, such as extremity, neurosurgery, and thoracic surgery, had no more than 10 patients among those with MCI and dementia status, so the surgery type was not shown in Table 1 due to the Centers for Medicare & Medicaid Services cell size suppression policy. The age of the patient significantly differed (*P <* 0.001) between the 3 categories with the average age of a patient with normal cognition being 74.8 years old, MCI being 77.9 years old, and dementia being the oldest average age at 81.1 years old. Those with more cognitive impairment were more likely to have outpatient surgery (*P <* 0.001). Seventeen percent of patients with dementia had inpatient surgery compared to 32% of patients with MCI and 37.4% of patients with normal cognition. Patients with dementia were also more likely to be non-Hispanic, non-white (NHNW) (33%), and Hispanic (16%) compared to patients with MCI (19.8% NHNW and 16.8% Hispanic) and normal cognition (8.2% NHNW and 5.3% Hispanic). Patients with cognitive impairment also had lower educational attainment, lower incomes, and were less likely to be married and living with a partner (*P <* 0.001 for all). There was no difference appreciated between groups with having an adult daughter (*P =* 0.549) Greater cognitive impairment was also associated with a higher Charleston comorbidity index (*P <* 0.001) and higher functional limitations (*P <* 0.001). Significant differences in anxiety, depression, arthritis, and other pain disorders between cognitive statuses were also found. In this cohort of patients, 40.6% of individuals had prior opioid exposure in the year before surgery; 7.7% had high-chronic opioid exposure; 21.0% had median-recent-intermittent opioid exposure; and 11.8% of patients had remote-intermittent opioid exposure.

### Initial Prescribing by Cognitive Status

Initial perioperative fill rates significantly differed by preoperative cognitive status with 58.1% of patients defined as having normal cognition, 54.4% of those with an MCI, and 33.5% of patients with dementia filled opioids before surgery (*P <* 0.001). Mean amount of opioids filled before surgery did not significantly differ between cognitive status groups (*P =* 0.678) (**Table 2**).

**TABLE 2.** Unadjusted Opioid Filled Rates, Refill Rates and Amount, and High-Risk Prescription Rates by Cognitive Status (n=1,874)

	Normal Cognitive Function (N = 1,274)		Mild Cognitive Impairment (MCI) (n = 400)		Dementia (n = 200)		
	Number	%	Number	%	Number	%	*P* value
Initial perioperative fill rate	740	58.1%	218	54.5%	67	33.5%	<0.001*
Amount of opioid (among patients who filled): Mean (SD)	601 (1508)		479 (604)		709 (1636)		0.678
30-day postoperative refill rate	307	24.1%	106	26.5%	42	21.0%	0.322
Amount of opioid filled within 30 days following surgery: mean (SD)	388 (1147)		255 (490)		313 (2304)		0.150
High risk prescribing rate†	701	55.0%	208	52.0%	66	33.0%	<0.001*

*Indicates statistically significant finding.

†High risk prescribing including any of the following: overlapping opioid prescriptions, overlapping opioid and benzodiazepine prescriptions, daily OME ≥100, long-acting opioid prescription, and filled opioid prescriptions from more than one prescriber.

**FIGURE 2.**
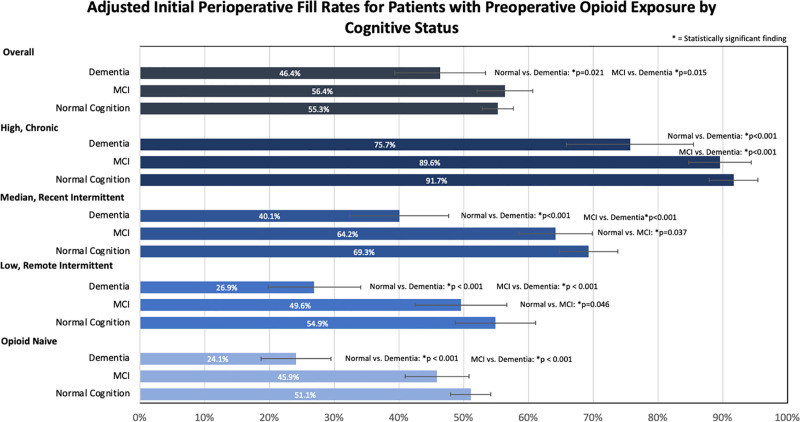
Adjusted initial perioperative fill rates for patients with preoperative opioid exposure by cognitive status.

When adjusted for patient factors, patients with dementia had a lower initial perioperative fill rate (46.4%) compared to patients with MCI (56.4%) (*P =* 0.015) and patients with normal cognition (55.3%) (*P =* 0.021). Patients in the high/chronic opioid exposure cohort had a significant difference in perioperative fill rates when comparing 91.1% for normal cognition vs 75.1% for dementia and 89.2% for MCI vs 75.1% for dementia, but no significant difference between normal cognition and MCI. For the medium/recent intermittent cohort and the low/remote cohort, all 3 cognitive statuses differed significantly from each other with regard to their perioperative opioid fill rates. For the medium/recent intermittent cohort, normal cognition had an initial preoperative fill rate of 69.2%, MCI was 64.2%, and dementia was 40.2%. For the low/remote cohort, normal cognition had an initial preoperative fill rate of 54.8%, MCI was 49.5%, and dementia was 26.9%. In the opioid-naive cohort, dementia (24.2%) compared to normal cognition (51.1%) and to MCI (46.0%) showed a statistically significant difference in perioperative fill rates (both *P <* 0.001), with perioperative fill rates being lower for dementia patients. There was not a significant difference between the normal cognition group and the MCI group in the opioid-naive cohort (**Fig. 2**). Analysis of the role of family support on opioid prescribing and cognitive status assessed using logistic regression adjusted for other variables revealed no significant interaction terms; geographic proximity to adult children, having a daughter, and marital status/living arrangement did not significantly affect initial perioperative opioid fill rate for different cognitive statuses.

### High Risk Prescribing by Cognitive Status

High-risk prescribing rates significantly differed between the 3 groups (*P <* 0.001). High-risk prescribing was at a rate of 55% in the normal cognition group, 52% in the MCI group, and 33% in the dementia group. All cause-readmission rates were significantly higher (*P =* 0.003) in the dementia group at 11.5%, compared to 5.4% in the normal cognition group and 7.8% in the MCI group (**Table 2**).

**FIGURE 3.**
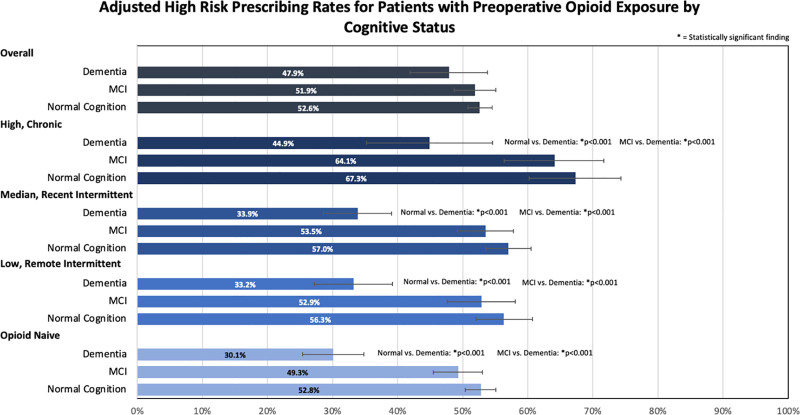
Adjusted high risk prescribing rates for patients with preoperative opioid exposure by cognitive status.

When high-risk prescribing rates were adjusted to take patient factors into account, patients with dementia had significantly lower risk prescribing than patients with normal cognition and MCI in all 4 preoperative opioid exposure cohorts (high/chronic, medium/recent intermittent, low/remote intermittent, and opioid naive) (*P <* 0.001). There was no significant difference in high-risk prescribing rates between patients with normal cognition and patients with MCI for all 4 preoperative opioid exposure cohorts (**Figure 3**).

(Supplemental Table S4, http://links.lww.com/AOSO/A236, which demonstrates the full model for adjusted odds ratios for filling Postoperative Opioid Prescriptions and Total Postoperative Opioid Prescriptions Amount among Cognitive Impairment Medicare Patients, by Preoperative Opioid Exposure).

### Refill Rate

**FIGURE 4.**
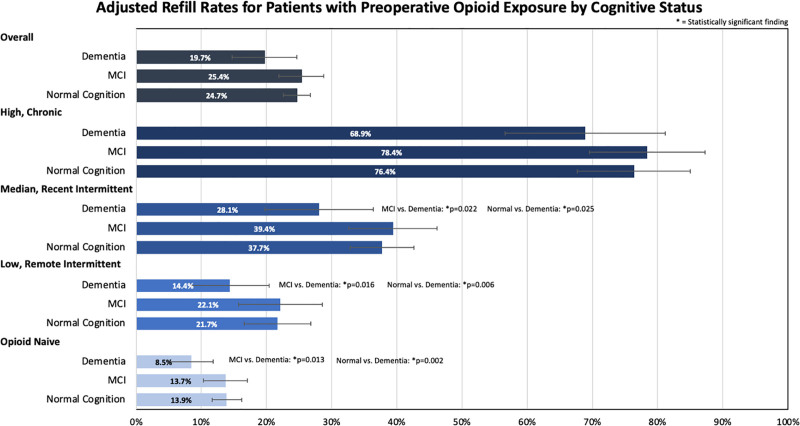
Adjusted refill rates for patients with preoperative opioid exposure by cognitive status.

When looking at unadjusted data, there was no significant difference in the 30-day postoperative refill rate between normal cognition, MCI, and dementia cohorts (*P =* 0.322) (**Table 2**). When adjusted for patient factors, there was not a significant difference between the 30-day postoperative refill rate and the mean amount of opioids filled within 30 days following surgery between the 3 cognitive status groups. The overall refill rate was 24.7% for normal cognition, 25.4% for MCI, and 19.7% for dementia. There was no statistically significant difference in the high/chronic opioid exposure cohort for postoperative refill rates between patients with normal cognition, MCI, and dementia. In the medium/recent intermittent, low/remote intermittent, and opioid-naive cohorts, opioid refill rates were found to be significantly lower for dementia patients vs both normal cognition and MCI. For these 3 cohorts, normal cognition and MCI did not have a significant difference in adjusted opioid refill rates (**Fig. 4**). Logistical regression analysis of the role of family support on opioid prescribing and cognitive status adjusted for other variables revealed no significant interaction terms; there was no significant difference in 30-day postoperative refill rate based on family support for different cognitive statuses.

### Adjusted Total Amount

**FIGURE 5.**
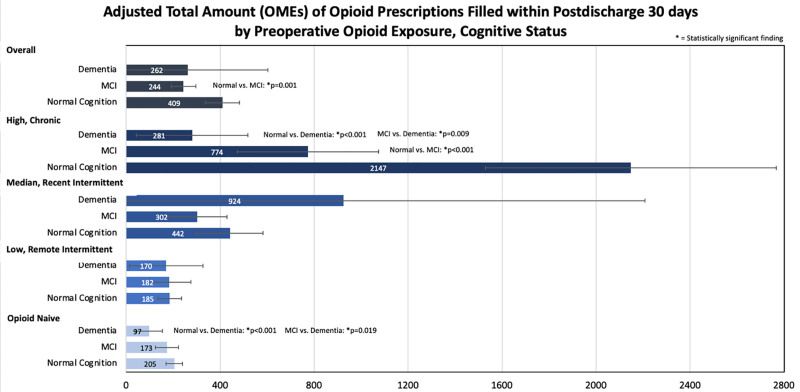
Adjusted total amount (OMEs) of opioid prescriptions filled within 30 days postdiscahrge by preoperative opioid exposure, cognitive status.

In the high/chronic cohort, adjusted total amount (OMEs) of the opioid prescription filled within 30 days postdischarge was significantly higher in patients with normal cognition (2147) compared to both patients with MCI (774) and patients with dementia (281) (*P <* 0.001 for both). Patients with dementia in the high chronic cohort had a significantly lower adjusted total amount of opioid prescriptions filled within 30 days postdischarge compared to patients with MCI (*P =* 0.009). No significant difference was found between patients with normal cognition, MCI, and dementia for the medium/recent intermittent preoperative opioid exposure cohort as well as the low/remote intermittent preoperative opioid exposure cohort. In the opioid-naive cohort, patients with dementia (97) had a significantly lower adjusted total amount (OMEs) of the opioid prescription filled within 30 days postdischarge compared to patients with normal cognition (205) and patients with MCI (173) (*P <* 0.01 and *P =* 0.019, respectively). In assessing all preoperative opioid exposure groups combined, only MCI patients (244) had significantly fewer opioid prescriptions filled within 30 days of discharge compared to patients with normal cognition (409), with a *P =* 0.001 (**Fig. 5**). Analysis of the role of family support on opioid prescribing and cognitive status assessed using logistic regression adjusted for other variables revealed no significant interaction terms; the total amount (OMEs) of opioid prescription filled within 30 days postdischarge is not significantly varied by family support for different cognitive status.

## DISCUSSION

In this cohort of older adults in the United States, we observed marked differences in opioid prescribing after surgery by cognitive status. For example, patients with dementia were less likely to receive initial prescriptions and refills after surgery and were less likely to be exposed to high-risk prescribing practices compared to patients with normal cognition. However, there were no overall differences observed in initial fill rates and refill rates among individuals with normal cognition and MCI. In addition, we observed differences in preoperative opioid status. Patients with normal cognition or MCI were more likely to present with preoperative opioid exposure compared with patients with dementia. Taken together, our findings suggest an opportunity to improve perioperative pain management that is contextualized to patient needs through further studies to understand the appropriateness of care and pain control in patients with normal cognition, MCI, and dementia.

Our findings align with previous research examining opioid use and pain management after surgery. In a study on postoperative opioid consumption and cognitive function in older adults with hip fracture, dementia was associated with less opioid use postoperatively.^19^ Additionally, in a retrospective observational study on postoperative pain management in patients with dementia in Japan, there was no difference in postoperative opioid use among patients with dementia compared with patients without dementia.^20^ While we cannot capture all factors that may differ between cohorts in our study, we did control for age, sex, race/ethnicity, income, marital status, and many other social factors, suggesting that a decreased initial prescription and refills could lead to undertreating pain in the MCI and dementia population.

Pain in dementia is often expressed through behavioral disturbances.^21^ These behavioral and psychological symptoms of dementia arising as a result of pain, such as agitation and aggression, can be extremely distressing for the individual and/or caregiver and possibly lead to the inappropriate prescribing of antipsychotic medication instead of adequate pain treatment.^21^ Additionally, in an observational study of pain assessments of patients with dementia, it was found that self-reporting alone was not sufficient to assess pain in elderly people with dementia and that pain is often ignored and undertreated in people with dementia.^22^ Although other studies have shown more opioid use in patients with cognitive impairment, some studies have shown that it is possibly more difficult to control pain in this population. In a 2019 study of opioid prescriptions in nursing home residents with advanced dementia in Norway, 19% were prescribed opioids for their pain, and of that percent, almost 80% of those prescribed opioids were still in pain.^23^ A 2022 study on pain and Alzheimer’s disease also using HRS data found that patients with dementia had a lower likelihood of reporting pain presence, lower reported pain severity, and lower likelihood of receiving pain treatment, supported the notion that patients with dementia may be undertreated for their pain.^24^

Alternatively, our findings could be interpreted in light of opioid-related harms, and be encouraging in that patients with dementia are less likely to be exposed to opioids in the perioperative period and high-risk prescribing. Prior research has shown increased harm with greater amounts of opioids prescribed. Santosa et al.^25^ found that the incidence of serious falls and fall related injuries increased with higher total amounts of opioids prescribed. Previous literature on high-risk prescribing found a 74% high-risk prescribing rate for chronic opioid users covered by private insurance between the ages of 18–64 years, a value higher than any of our cognitive status groups.^26^ Conversely, Santosa et al.^27^ utilized Medicare claims data and found a value of 5.1% high-risk prescribing among opioid-naive Medicare patients who underwent major/minor surgery, much lower than the high-risk prescribing rates of any of our cognitive groups. There is a fine balance between pain management and simultaneously mitigating other risks associated with opioid use in older adults, a population more at risk for fall-related injuries to begin with.

We observed lower preoperative and postoperative opioid fill rates among patients with cognitive impairment, which could have benefits if it is assumed that pain control was satisfactory. It is possible the best strategy for pain treatment may be leaning towards less opioid use in patients with MCI and dementia, as this patient population is at higher risk for adverse outcomes from postoperative opioid use. For example, less prescribing could mitigate the risk of prolonged use, as well as the risk of overdose related to opioids if barriers exist in understanding medication risks or the potential risk of polypharmacy. Polypharmacy is more common among patients with dementia than patients without dementia^28^ and polypharmacy at dementia diagnosis is associated with a higher risk of adverse health outcomes.^29^ These studies support that the more prescriptions a patient has to take, the more likely they may not take it correctly. Less opioid use before and after surgery in patients with MCI and dementia could also decrease the risk of delirium. Dementia is an important risk factor for delirium and is common in frail elderly. Patients with dementia who have a hip fracture with surgical repair were more likely to experience postoperative delirium than those without dementia. Although it was found that there was no difference in 6-month mortality in delirious patients with or without dementia.^30^

### Limitations

Our study has several notable limitations. Cognitive status was assessed at a median of 8 months before surgery, which may have not aligned with their cognitive status on the day of surgery. For example, patients could have been placed into MCI and subsequently progressed to dementia during the time between their initial assessment and index procedure. Although cognitive assessments were performed by trained staff, misclassification of patients into the wrong cognitive group is possible as HRS assesses cognition based on a limited set of cognitive tests or proxy assessments without a full clinical evaluation.^31^ In addition, our measured outcomes were based on how much opioid was filled before or after surgery, which does not necessarily indicate the actual consumption of the opioid. For example, the patient fills prescription but does not consume any or opioids were obtained outside of those prescriptions. Finally, this data was collected from patients 65+ and may not be generalizable to other age groups. Although this data being collected from 2007 to 2016 may not represent current prescribing, the data allow us to study the relationship between cognitive status and opioid prescribing among surgical patients.

### Implications moving forward

Despite the above limitations, this study has important implications for patients with cognitive impairment and dementia who are prescribed opioids before or after a surgical procedure. Due to the higher risk of falls and fall-related injuries in patients prescribed opioids postoperatively, it is important for physicians to assess the cognitive status of patients when considering postoperative pain control options. Various methods currently exist in the primary care setting for assessing cognitive function in older adults, which could be extended to the perioperative space. For example, the 3 most common cognitive assessment tools are the Mini-Mental State Examination, the Montreal Cognitive Assessment, and the Mini-cog test. In addition to these cognitive assessments, surgeons should be aware of other potential risk factors for cognitive impairment such as hypothyroidism, hypercalcemia, hyperglycemia, and vitamin B12 deficiency; all of which can be common among surgical patients.^32^

It is also important to consider opioid alternatives for patients with MCI or dementia to reduce the risk of adverse events. Acetaminophen (paracetamol) and nonsteroidal anti-inflammatory drugs are the two medications most commonly used in combination with opioids and can reduce opioid use when combined with other options.^33^ These protocols are common among recent multimodal analgesia regimens and enhanced recovery pathways to reduce opioid use and minimize the side effects of individual drugs.^34^

Finally, the creation of preoperative screening tools that assess opioid use can guide postoperative opioid treatment. This may include an assessment of the cognitive status of a patient and have that factor into their decision for pain control postoperatively. Chronic opioid use for chronic pain or exposure to opioids before surgery could be added to the preoperative clearance that patients receive either from their primary care physician or from anesthesia in the same way patients are cleared from a cardiac standpoint for surgery. A randomly selected 100-patient chart review at the University of Utah found that 93% of patient’s charts lacked preoperative opioid screening and an in-depth review of the chart found that one-third of these patients were using opioids before surgery.^35^ This discrepancy in screening could result in negative postoperative outcomes for the patient. A standardized preoperative opioid assessment for all patients could help mitigate this risk.

## CONCLUSIONS

Patients with preexisting dementia before having surgery fill and refill their opioid prescriptions at a lower rate compared to patients with normal cognition or a mild cognitive impairment. A cognitive assessment is an additional tool surgeons can use to determine a patient’s individualized postoperative pain control plan. It is important to assess both the patient ability to report pain to ensure it is not being undertreated as well as how potentially higher doses of opioid will negatively impact a patient with a cognitive impairment’s function.

## Supplementary Material


