# Supplementary material for: The Association of Cognitive Status and Post-Operative Opioid Prescribing in Older Adults
Source: Ann Surg Open. 2023 Aug 21;4(3):e320. doi: 10.1097/AS9.0000000000000320 (PMC10513135; doi:10.1097/AS9.0000000000000320)
Supplement: Supplementary file 1 [file as9-4-e320-s001.pdf]

Table S1: CPT-4 Codes: Current Procedural Terminology Fourth Edition (CPT-4) codes included in analysis

**Surgery Type**	**Elective/Non-elective/Either**	CPT-4 Codes
Abdominal	Elective	38120, 38780, 43610, 43611, 43620, 43621, 43622, 43640, 43644, 43645, 43770, 43771, 43772, 43773, 43832, 43840, 43843, 43845, 43846, 43847, 43848, 44110, 44213, 44312, 44314, 44340, 44345, 44346, 44615, 44950, 44970, 45000, 45005, 45020, 45160, 45190, 45400, 45560, 46040, 46045, 46060, 46700, 46750, 47010, 47120, 47122, 47125, 47130, 47370, 47380, 47480, 47740, 47741, 47785, 48100, 48120, 48150, 48152, 48153, 48154, 48155, 48540, 48548, 49021, 49203, 49204, 49205, 49250, 49255, 49321, 49322, 49323, 49324, 49325, 49421, 49505, 49520, 49540, 49550, 49555, 49560, 49561, 49565, 49566, 49568, 49570, 49585, 49653, 60540, 60545, 60650
	Non-elective	43870, 44021, 44050, 44055, 44900, 44955, 49020, 49040, 49060, 49402, 49507, 49521, 49525, 49553, 49557, 49572, 49587
	Either	20102, 38100, 43500, 43501, 43631, 43632, 43633, 43634, 43774, 43800, 43820, 43825, 43850, 43860, 43880, 43886, 43888, 44005, 44010, 44020, 44025, 44120, 44121, 44125, 44130, 44139, 44140, 44141, 44143, 44144, 44145, 44146, 44147, 44150, 44151, 44155, 44156, 44157, 44158, 44160, 44180, 44187, 44188, 44202, 44204, 44205, 44206, 44207, 44208, 44210, 44211, 44212, 44227, 44310, 44320, 44602, 44603, 44604, 44605, 44620, 44625, 44626, 44640, 44650, 44661, 44800, 44820, 44850, 44960, 45110, 45111, 45112, 45113, 45114, 45119, 45123, 45130, 45395, 45397, 45402, 45505, 45540, 45541, 45550, 46947, 47420, 47460, 47560, 47562, 47563, 47564, 47600, 47605, 47610, 47612, 47620, 47760, 47780, 48105, 48140, 48145, 48510, 48520, 49000, 49002, 49010, 49422, 49425, 49590, 49650, 49651, 49652, 49654, 49655, 49656, 49657, 49900

Breast	Elective	11960, 19020, 19110, 19120, 19125, 19126, 19260, 19300, 19301, 19302, 19303, 19304, 19305, 19307, 19316, 19318, 19324, 19325, 19328, 19340, 19342, 19350, 19357, 19361, 19367, 19370, 19371, 19380
	Non-elective	
	Either	
Cardiac	Elective	33266, 33517, 33518, 33519, 33521, 33533
	Non-elective	33025
	Either	33405, 33406, 33410, 33426, 33427, 33430, 33534, 33535, 33860, 33863
Extremity	Elective	25076, 25077, 25111, 25112, 26350, 26356, 26440, 26445, 27047, 27048, 27049, 27303, 27327, 27328, 27329, 27590, 27592, 27596, 27880, 27881, 27886, 38740, 38745, 38760, 38765, 64708, 64831
	Non-elective	26410, 26418, 26540, 27600, 27602
	Either	20103, 26615, 26727, 26735, 26952, 27301, 27594, 27603, 27882, 27884, 27888, 28003, 28800, 28805
Gynecologic	Elective	56405, 56420, 56440, 56630, 57120, 57135, 57260, 57267, 57280, 57283, 57425, 57522, 57530, 58145, 58150, 58152, 58180, 58200, 58210, 58260, 58262, 58270, 58290, 58291, 58543, 58544, 58545, 58546, 58548, 58550, 58552, 58553, 58554, 58570, 58571, 58572, 58573, 58950, 58951, 58952, 58953, 58954, 58956, 58957, 59150, 59151
	Non-elective	
	Either	56620, 56740, 57240, 57250, 57265, 57268, 57282, 57285, 57288, 57295, 57300, 58140, 58146, 58541, 58542, 58700, 58720, 58740, 58925, 58940, 58943, 59120, 59121
Head/neck/face	Elective	21235, 38542, 38570, 38720, 38724, 41112, 41113, 41120, 41155, 42120, 42140, 42410, 42415, 42420, 42426, 42821, 42826, 60210, 60212, 60220, 60225, 60252, 60254, 60500, 60505, 69632, 69633, 69642, 69645, 69646
	Non-elective	21356, 21365, 21462, 42962

	Either	21461, 31360, 31365, 31780, 41110, 41135, 42145, 42440, 42700, 42815, 42831, 60200, 60240, 60260, 60270, 60271, 60280, 60502, 69631
Hip replacement, total and partial	Elective	27130, 27132
	Non-elective	
	Either	
Knee arthroplasty	Elective	27438, 27442, 27445, 27446, 27447
	Non-elective	
	Either	
Neurosurgical	Elective	61500, 61512, 61518, 61536, 61548, 61597, 61700, 61885, 61886, 61888
	Non-elective	
	Either	61304, 61312, 61313, 61315, 61343, 61458, 61510
Shoulder	Elective	23470, 23472
	Non-elective	
	Either	
Soft tissue	Elective	11044, 14301, 15220, 15240, 15260, 15620, 15630, 15731, 15732, 15830, 15937, 20005, 20926
	Non-elective	11004, 11005, 11006
	Either	10180, 11000, 11008, 11042, 11043, 15734, 15738, 15931, 15936, 15946
Spine	Elective	21930, 22523, 22524, 22532, 22533, 22534, 22548, 22551, 22552, 22554, 22556, 22558, 22585, 22586, 22590, 22595, 22600, 22610, 22612, 22614, 22630, 22632, 22633, 22634, 22800, 22802, 22804, 22808, 22810, 22812, 22852, 22856, 22857, 22858, 22859, 22861, 22864, 63020, 63030, 63046, 63075, 63276, 64713
	Non-elective	22015, 22830
	Either	63001, 63005, 63012, 63015, 63017, 63042, 63045, 63047, 63048, 63056, 63081, 63267

Thoracic	Elective	21615, 32141, 32651, 32655, 39400, 39540, 39541, 43107, 43112, 43117, 43122, 43130, 43279, 43280, 43281, 43282, 43330, 43332, 60520
	Non-elective	21750
	Either	21501, 32100, 32220, 32225, 32320, 32480, 32482, 32484, 32650, 32652, 43325, 43415
Urologic	Elective	50220, 50230, 50234, 50240, 50542, 50543, 50544, 50545, 50546, 50548, 51500, 51590, 51595, 51596, 51992, 52234, 52235, 52240, 52320, 52344, 52450, 52500, 52630, 52640, 52647, 52648, 53440, 53445, 54530, 54640, 54840, 55040, 55041, 55100, 55530, 55535, 55810, 55815, 55840, 55842, 55845, 55866, 55873
	Non-elective	50060, 54600
	Either	51040, 51050, 51840, 51845, 52601, 54520, 54522, 54860, 55110
Vascular	Elective	35390, 35691, 35694, 35907, 36475, 36476, 36478, 36838, 37607, 37700, 37718, 37722, 37760, 37765, 37766, 37780, 60600, 60605
	Non-elective	34101, 34111, 34490, 35045, 35082, 35092, 35103, 35132, 35226, 35286, 35700, 35875, 35876
	Either	33877, 33880, 33881, 34001, 34151, 34201, 34203, 34421, 34800, 34802, 34803, 34804, 34805, 34812, 34825, 34830, 34831, 34900, 35001, 35011, 35081, 35091, 35102, 35121, 35131, 35141, 35142, 35151, 35190, 35201, 35206, 35221, 35236, 35256, 35281, 35301, 35302, 35303, 35304, 35305, 35321, 35331, 35351, 35355, 35361, 35371, 35372, 35471, 35501, 35506, 35521, 35525, 35539, 35540, 35556, 35558, 35560, 35565, 35566, 35571, 35583, 35585, 35587, 35601, 35606, 35621, 35626, 35631, 35637, 35638, 35646, 35647, 35654, 35656, 35661, 35665, 35666, 35671, 35721, 35741, 35761, 35800, 35840, 35860, 35879, 35881, 35883, 35884, 35903, 37205, 37215, 37220, 37221, 37224, 37225, 37226, 37227, 37228, 37229, 37230, 37617, 37618
