# Supplementary material for: The Association of Cognitive Status and Post-Operative Opioid Prescribing in Older Adults
Source: Ann Surg Open. 2023 Aug 21;4(3):e320. doi: 10.1097/AS9.0000000000000320 (PMC10513135; doi:10.1097/AS9.0000000000000320)
Supplement: Supplementary file 2 [file as9-4-e320-s002.pdf]

Table S2: Patient Groups and Attributes of Preoperative Opioid Exposure Within One Year Before Surgery

			Total Oral Morphine Equivalent (OME) Filled During Preop		Duration in Month		Continuity in Month		Recency in Month	
Group	n	%	Median	Lower - Upper Quartile	Median	Lower - Upper Quartile	Median	Lower - Upper Quartile	Median	Lower - Upper Quartile
Naïve	1113	59.4%								
Low, Remote Intermittent	222	11.8%	225	129 - 600	1	1 - 1	1	1 - 1	8	6 - 10
Medium, Recent Intermittent	394	21.0%	735	300 - 1840	2	1 - 4	1	1 - 2	2	1 - 3
High, Chronic	145	7.7%	8790	5400 - 17400	10	8 - 11	8	6 - 11	2	1 - 2
