# Supplementary material for: The Association of Cognitive Status and Post-Operative Opioid Prescribing in Older Adults
Source: Ann Surg Open. 2023 Aug 21;4(3):e320. doi: 10.1097/AS9.0000000000000320 (PMC10513135; doi:10.1097/AS9.0000000000000320)
Supplement: Supplementary file 3 [file as9-4-e320-s003.pdf]

Table S3. Pain diagnoses

Pain Disorder	Diagnosis Code
Back	**ICD-9:** 722.30, 722.32, 722.33, 722.70, 722.72, 722.73,722.80, 722.82, 722.83, 722.90, 722.92, 722.93,737.1, 737.3, 738.4, 738.5, 739.2, 739.3, 739.4,756.10, 756.11, 756.12, 756.19, 805.4, 805.8,839.2, 839.42, 846, 846.0, 847.1, 847.2, 847.3,847.9, 721.3* - 721.9*, 722.2*, 724.*, 756.13<br>**ICD-10**: M40.0*,M40.2*,M41.00,M41.04-M41.08, M41.20, M41.24-M41.27,M41.3*,M41.80,M41.84-M41.87,M41.9,M43.00, M43.04-M43.10, M43.14-M43.19,M43.27,M43.28,M46.40, M46.44-M46.48, M47.10, M47.14-M47.16, M47.814-M47.819, M48.00,M48.04-M48.08,M48.10, M48.14-M48.20, M48.24-M48.27,M48.30,M48.34-M48.38,M48.9,M51.04-M51.06, M51.44-M51.47, M51.84-51.87,M51.9,M53.2*4-M53.2*9, M53.3, M53.9, M54.04-M54.08, M54.14-M54.18, M54.3*-M54.6, M54.89, M54.9, M96.1-M96.3, M96.5, M99.02-M99.04, M99.83, M99.84, Q76.2,Q76.4*, S22.009A,S23.101A, S23.111A, S23.121A, S23.123A, S23.131A,S23.133A,S23.141A, S23.143A, S23.151A, S23.153A, S23.161A,S23.163A,S23.171A, S23.3XXA, S23.8XXA, S23.9XXA, S32.009A, S32.10XA, S32.2XXA, S33.101A, S33.2XXA, S33.5XXA, S33.6XXA, S33.8XXA, S33.9XXA
Neck	**ICD9:** 721.0*, 721.1*, 722.0*, 722.31, 722.71, 722.81,722.91, 723.*, 839.0, 839.1, 847.0<br>**ICD-10:** M41.02,M41.03,M41.22,M41.23,M41.82, M41.83, M43.01, M43.02, M43.03, M43.11, M43.12, M43.13, M43.6, M47.11, M47.12, M47.13, M47.811, M47.812, M47.813, M48.01, M48.02, M48.03, M50.0*, M50.2*, M50.8*, M50.9*, M53.0, M53.1, M53.82, M54.01, M54.02, M54.03, M54.11, M54.12, M54.13, M54.2, M99.01, S13.101A, S13.111A, S13.121A, S13.131A, S13.141A, S13.151A, S13.161A, S13.171A, S13.181A, S13.4XXA, S13.8XXA
Arthritis and joint pain	**ICD-9:** 711.*, 712.*, 713.*, 714.*, 715.*,716.*, 717.*, 718.*, 719.*, 725,726.*, 727.*, 728.*, 729.3*, 729.7*,729.8*, 729.9*, 730.*, 731.*, 732.*,733.*, 734, 735.*, 736.*, 737.2*,737.4*, 738.1*, 710, 710.1, 710.3, 710.4,710.5, 710.8, 710.9, 729, 729.2, 729.4, 729.5,729.6, 737, 737.8, 737.9, 738, 738.2, 738.3,738.6, 738.7, 738.8, 738.9, 739, 739.1, 739.5,739.6, 739.7, 739.8, 739.9<br>**ICD-10:** M00.*,M01.*,M02.*,M05.*,M06.*,M08.*,M11.*,M12.*, M13.*, M14.*, M15.*, M16.*, M17.*, M18.*, M19.*, M20.*,M21.*,M22.*,M23.*, M24.*,M25.* ,M32.*, M33.*, M34.*, M36.*, M42.*, M61.*, M62.*, M65.*, M66.*, M67.*, M70.*, M71.*, M72.*, M75.*, M76.*, M77.*, M80.*, M81.*, M84.*, M85.*,M86.*,M87.*,M88.*,M89.*,M90.*, M91.*,M92.*,M93.*, M94.*,M95.*,M40.1*,M40.4*,M40.5*,M41.4*,M41.5*,M43.8*,M60.0*,M60.1*, M60.2*,M79.6*,M43.9*,M35.1*,M35.2*,M35.3*,M35.4*,M35.5*,M35.6*, M35.7*, M35.8*,M35.9*,M96.4*,R25.2*,R26.2*,R29.4*,R29.898, S12.000K, S12.001K,S12.100K,S12.101K,S12.200K,S12.201K,S12.300K, S12.301K, S12.400K,S12.401K,S12.500K,S12.501K,S12.600K, S12.601K, S42.009K, S42.009P,S42.209K,S42.209P,S42.91SK, S42.92SK, S82.009P, S82.009Q, S82.009R, S92.819K,S92.819P, S92.909K,S92.909P,S92.919K, S92.919P, S99.209K, S99.209P, S99.219K, S99.219P, S99.229K, S99.229P, S99.239K, S99.239P, S99.249K,S99.249P,S99.299K,S99.299P,M48.40*A,M48.41*A, M48.42*A,M48.43*A,M48.44*A,M48.45*A,M48.46*A,M48.47*A, M48.48*A, M48.50*A,M48.51*A,M48.52*A,M48.53*A,M48.54*A, M48.55*A, M48.56*A, M48.57*A,M48.58*A, S02.91*K,S02.92*K, S42.90*K, S52.90*K,S62.90*K,

	S72.90*K,S82.90*K,S52.90*M, S72.90*M,S82.90*M,S52.90*N, S72.90*N, S82.90*N, S52.90*Q, S72.90*Q,S82.90*Q,S52.90*R,S72.90*R, S82.90* R,S42.90*P, S42.91*P,S42.92*P,S52.90*P,S62.90*P,S72.90*P,S82.90*P, S22.9**K,S32.9**K
Other Pain Disorders	**ICD-9:** 346.00, 346.01, 346.02, 346.03, 346.10, 346.11, 346.12, 346.13, 346.20, 346.21, 346.22, 346.23, 346.30, 346.31, 346.32, 346.33, 346.40, 346.41, 34642, 346.43, 346.50, 346.51, 346.52, 346.53, 346.60, 346.61, 346.62, 346.63, 346.70, 346.71, 346.72, 346.73, 346.80, 346.81, 346.82, 346.83, 346.90, 346.91, 346.92, 346.93, 524.60, 524.61, 524.62, 524.63, 524.64, 524.69, 307.81, 784.0, 723.8, 375.15, 729.1, 786.59, 530.5, 595.1, 536.8, 564.*, 617.*, 625.7*, 710.2, 388.3, 780.71, 780.52<br>**ICD-10:** G43.*,R51.*,K58.*,K59.*,N80.*,K30.*,M60.9*, M79.1*,M79.9*, M60.8*, K22.4*,M26.6*,H93.1*,M35.0*,M54.81*,N30.10*, N30.11*,R07.82*, R07.89*,N94.81*,R53.82*,G47.00*,H04.129,G44.201, G44.209

ICD-9, ICD-10 = International Statistical Classification of Diseases and Related Health Problems, Ninth/Tenth Edition.
