# Supplementary material for: The Association of Cognitive Status and Post-Operative Opioid Prescribing in Older Adults
Source: Ann Surg Open. 2023 Aug 21;4(3):e320. doi: 10.1097/AS9.0000000000000320 (PMC10513135; doi:10.1097/AS9.0000000000000320)
Supplement: Supplementary file 4 [file as9-4-e320-s004.pdf]

Full Model for Opioid Prescribing

Table S4: Adjusted Odds Ratios for Filling Postoperative Opioid Prescriptions and Total Postoperative Opioid Prescriptions Amount among Cognitive Impairment Medicare Patients, by Preoperative Opioid Exposure

	Filled Initial Prescription		Refill w/i 30 days		High Risk Prescribing w/i 30 days		Total OMEs Filled w/i 30 days		All-cause Readmission w/i 30 days	
	aOR (95%CI)	p-value	aOR (95%CI)	p-value	aOR (95%CI)	p-value	Coef.	p-value	aOR (95%CI)	p-value
Cognitive Status (ref: Normal)										
Mild Cognitive Impairment (MCI)	1.06 (0.81 1.38)	0.673	1.06 (0.75 1.49)	0.745	0.93 (0.65 1.34)	0.693	-42 (-112 29)	0.251	1.17 (0.73 1.89)	0.519
Dementia	0.63 (0.42 0.93)	0.020	0.64 (0.37 1.11)	0.111	0.64 (0.35 1.15)	0.135	-146 (-312 21)	0.086	1.50 (0.84 2.67)	0.170
Preoperative Opioid Exposure (ref: Opioid Naïve)										
Low, Remote Intermittent	1.20 (0.87 1.67)	0.268	1.94 (1.28 2.94)	0.002	1.39 (0.89 2.18)	0.153	-20 (-84 44)	0.544	0.99 (0.53 1.83)	0.964
Median, Recent Intermittent	2.54 (1.92 3.37)	<0.001	5.03 (3.67 6.89)	<0.001	1.48 (1.02 2.15)	0.039	237 (84 389)	0.002	1.07 (0.65 1.77)	0.791
High, Chronic	15.94 (9.05 28.09)	<0.001	33.85 (19.41 59.05)	<0.001	4.02 (2.02 7.97)	<0.001	1942 (1322 2562)	<0.001	2.07 (1.10 3.92)	0.025
Interaction bw Cognitive Status & Preoperative Opioid Exposure										
MCI & Low, Remote Intermittent							29 (-87 145)	0.627		
MCI & Median, Recent Intermittent							-108 (-306 90)	0.284		
MCI & High, Chronic							-1341 (-2037 -645)	<0.001		
Dementia & Low, Remote Intermittent							93 (-78 264)	0.286		
Dementia & Median, Recent Intermittent							590 (-679 1860)	0.362		
Dementia & High, Chronic							-1758 (-2418 -1098)	<0.001		
Surgery Type (ref: Abdominal)										
Breast	1.38 (0.86 2.19)	0.182	1.07 (0.58 1.97)	0.873	0.81 (0.43 1.50)	0.498	72 (-78 222)	0.349	0.17 (0.04 0.72)	0.016
Cardiac	1.32 (0.58 3.00)	0.513	2.61 (1.09 6.25)	0.032	4.75 (1.31 17.22)	0.018	161 (-81 403)	0.191	1.96 (0.69 5.59)	0.208
Extremity	0.67 (0.31 1.43)	0.300	0.87 (0.32 2.32)	0.778	1.09 (0.38 3.17)	0.871	-17 (-258 225)	0.893	0.88 (0.25 3.12)	0.843
Gynecologic	0.94 (0.57 1.56)	0.822	0.60 (0.28 1.27)	0.182	0.57 (0.29 1.11)	0.098	49 (-137 234)	0.606	0.39 (0.12 1.33)	0.133
Head/Neck/Face	0.64 (0.36 1.15)	0.139	1.20 (0.57 2.54)	0.637	0.42 (0.20 0.90)	0.026	-50 (-203 103)	0.519	0.78 (0.26 2.34)	0.856
Hip Replacement, Total, Partial	3.72 (0.82 16.79)	0.088	2.03 (0.67 6.19)	0.213	2.37 (0.36 15.55)	0.37	234 (30 437)	0.024	*	*
Knee Replacement	2.27 (1.02 5.06)	0.046	18.54 (8.87 38.75)	<0.001	2.79 (0.90 8.67)	0.076	516 (319 714)	<0.001	0.32 (0.04 2.39)	0.265
Neurosurgery	0.47 (0.10 2.18)	0.334	4.48 (0.81 24.70)	0.085	6.10 (0.87 42.94)	0.069	194 (-25 413)	0.083	2.33 (0.26 20.65)	0.448
Shoulder Surgery	1.57 (0.50 4.93)	0.442	6.69 (2.32 19.23)	<0.001	1.28 (0.27 6.06)	0.757	198 (-67 463)	0.143	0.57 (0.07 4.51)	0.595
Soft Tissue	0.16 (0.11 0.24)	<0.001	0.81 (0.51 1.28)	0.367	0.42 (0.25 0.69)	0.001	-88 (-237 61)	0.248	0.63 (0.35 1.11)	0.109
Spine	1.59 (0.97 2.61)	0.067	3.94 (2.38 6.50)	<0.001	0.67 (0.36 1.24)	0.206	167 (-74 408)	0.174	0.65 (0.27 1.53)	0.319
Thoracic	1.27 (0.57 2.83)	0.555	0.73 (0.21 2.58)	0.631	0.78 (0.26 2.36)	0.662	100 (-27 228)	0.123	1.83 (0.59 5.71)	0.298
Urologic	0.58 (0.38 0.88)	0.011	1.06 (0.60 1.88)	0.840	0.63 (0.35 1.13)	0.121	-38 (-193 118)	0.643	0.29 (0.10 0.85)	0.024
Vascular	0.26 (0.18 0.37)	<0.001	0.74 (0.45 1.21)	0.230	0.40 (0.24 0.66)	<0.001	98 (-154 349)	0.447	0.64 (0.34 1.21)	0.169
Non-elective Surgery (ref: Elective)	0.73 (0.50 1.07)	0.105	1.40 (0.86 2.26)	0.174						
Inpatient (ref: Outpatient)					1.64 (1.16 2.33)	0.005				
Amount of Perioperative Opioid			1.00 (1.00 1.00)	0.001						
Filled Initial Prescription (ref: No)					47.79 (34.72 65.78)	<0.001			0.70 (0.45 1.07)	0.102
Age	0.97 (0.96 0.99)	<0.001	0.98 (0.96 1.00)	0.015			-9 (-16 -2)	0.016	1.02 (0.99 1.05)	0.125
Sex: Female (ref: Male)	0.78 (0.60 1.00)	0.047			0.83 (0.60 1.15)	0.264	-104 (-245 37)	0.149		
Race/Ethnicity (ref: Non-hispanic White)										
Non-hispanic Non-white							-14 (-226 198)	0.896		
Hispanic							-203 (-384 -21)	0.029		
Income (quartiles) (ref: 29,505 - 57,300)										
≤14,644			1.14 (0.76 1.70)	0.525			186 (-65 436)	0.147	0.73 (0.42 1.24)	0.239
14,645 - 29,504			1.23 (0.84 1.79)	0.285			-71 (-154 120)	0.248	0.78 (0.46 1.31)	0.347
>57,300			0.83 (0.56 1.22)	0.342			-68 (-183 47)	0.245	0.62 (0.35 1.13)	0.117
Marital status/living arrangement (ref: Married/partner)										
Unmarried/living with other	1.74 (1.21 2.51)	0.003					-128 (-310 54)	0.167		
Unmarried/living alone	0.96 (0.75 1.23)	0.761					-17 (-154 120)	0.805		
Have an adult daughter										
Charlson Comorbidity Index	0.94 (0.91 0.98)	0.004	1.04 (0.99 1.09)	0.089	1.06 (1.01 1.12)	0.027			1.14 (1.07 1.20)	<0.001
Functional limitations			1.04 (0.98 1.11)	0.214	1.06 (0.99 1.14)	0.092	27 (2 52)	0.031		
CES-D depressive symptoms (ref: 0)										
1-4			1.20 (0.88 1.62)	0.249					1.36 (0.87 2.10)	0.173
5-8			2.05 (1.34 3.14)	0.001					1.45 (0.79 2.67)	0.228
Tobacco Use (ref: No)							107 (-21 235)	0.100		
Anxiety Disorder	0.72 (0.51 1.02)	0.065			1.39 (0.87 2.23)	0.168	84 (-58 226)	0.248		
Depression	1.41 (1.02 1.94)	0.038			0.78 (0.52 1.19)	0.252	-99 (-238 39)	0.160		
Back Pain					0.77 (0.65 1.07)	0.116				
Neck Pain							167 (-107 441)	0.233		
Year (2007, 2008m 2009,……2016)	0.97 (0.93 1.00)	0.063	0.95 (0.91 1.00)	0.052			-14 (-30 2)	0.092		
c-statistics	0.780		0.856		0.917				0.717	

*Zero hip relpacement cases were readmitted in 30 days
